# Response to letter to editor “On ‘Bioinformatics in Russia: history and present-day landscape’ by M.A. Nawaz, I.E. Pamirsky, and K.S. Golokhvast” by Mikhail Gelfand

**DOI:** 10.1093/bib/bbaf172

**Published:** 2025-05-23

**Authors:** Muhammad Amjad Nawaz, Igor Eduardovich Pamirsky, Kirill Sergeevich Golokhvast

**Affiliations:** Advanced Engineering School (Agrobiotek), National Research Tomsk State University, Lenin Ave, 36, 634050 Tomsk, Tomsk Oblast, Russia; Centre for Research in the Field of Materials and Technologies, National Research Tomsk State University, Lenin Ave, 36, 634050 Tomsk, Tomsk Oblast, Russia; Advanced Engineering School (Agrobiotek), National Research Tomsk State University, Lenin Ave, 36, 634050 Tomsk, Tomsk Oblast, Russia; Siberian Federal Scientific Centre of Agrobiotechnology, Centralnaya, Presidium, 633501 Krasnoobsk, Russia; Advanced Engineering School (Agrobiotek), National Research Tomsk State University, Lenin Ave, 36, 634050 Tomsk, Tomsk Oblast, Russia; Siberian Federal Scientific Centre of Agrobiotechnology, Centralnaya, Presidium, 633501 Krasnoobsk, Russia

**Keywords:** bioinformatics, OMICs, Russia

##  

**Dear Editor,** 

Thank you for sharing the critique of our recent article [1]. We appreciate the opportunity to have our work carefully examined and critically engaged with. We are pleased that Respected Professor Mikhail Gelfand acknowledges that the ‘topic is clearly important,’ a recognition that aligns with the authors’ commitment, having worked on the manuscript for over a year. While the critique primarily concerns the inclusion and omission of institutional and researcher names, as well as the selection of surveyed literature, we would like to emphasize that the conclusions established in our article remain consistent and valid. Moreover, we recognize that literature selection in review articles can sometimes lead to differing opinions, and it is not uncommon for critiques to arise when certain works are not referenced. However, our selection was guided by thematic relevance and the overall coherence of the article, rather than the inclusion or omission of any particular work, or detailing individual or institutional contribution. Additionally, when writing a review article, authors must also adhere to the publisher’s requirements regarding article length. Below we respond to the comments presented in critique.

The industries mentioned—biotechnology, information technology, pharmaceuticals, and agriculture—are globally recognized as key drivers of bioinformatics research, as analyzed in several studies [2–4]. The data presented on these four industries is valid, relevant, up-to-date, and systematically organized. This was also acknowledged in comments by two of the four anonymous reviewers. Is such information available in one place elsewhere? On a similar note, critique mentions the role of president’s decree on the establishment of the Russian Science Foundation (RSF) and bonuses to researches, in relation to the observed increase in the number of publications by Russian researchers. This explanation strongly aligns with our statement. Why is that? Because the RSF is a major funding agency in the country, and it is understandable that it requires the project winners to publish in journals indexed in Web of Science or Scopus, Russian Science Citation Index, and other foreign bibliographic databases. For reference, the editor is referred to the ongoing project competitions and read the notification (in Russian ‘
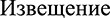
’) documents (https://rscf.ru/contests/) of any project call. Hence, the RSF’s establishment triggered the increase in the number of publications.

Regarding historical aspects, we appreciate the extended list of researchers and institutes. As editor will note, we had already provided reference [5] (no. 37 in the published article) in our article to guide the readers toward relevant historical details including conferences. Given that each institute mentioned has a rich history encompassing multiple dimensions of biological research, simply listing them would not have been sufficient. By mentioning these institutes and providing their website links, we have ensured that readers who wish to explore their histories further have the necessary resources. Similarly, Information Box II, when offering general definitions or addressing global aspects, naturally includes relevant citations from authors affiliated with institutions outside Russia. Moreover, this box briefly covered translational regulatory networks, whereas in text, we highlighted other key directions of the leading institute(s) as well. Our goal was to highlight the general research direction and trends, rather than to review every individual publication from these institutes. Regarding Skoltech, we specifically stated: “The facility is involved in using sequencing technologies to solve agricultural and health-related problems…”. At no point did the authors claim that the institute or facility is sequencing 100 000 human genomes or provides “bioinformatics service”. We included the statement on 100 000 genomes sequencing (https://www.biotechcampus.ru/; last accessed on 27 March, 2025) here only because it aligns with the fact that the institute itself has published relevant human genome projects (reference no. 57 in published article). Moving further, the comments on protein research largely emphasize historical and notable contributions in the field and misconceived our statement: “At the level of collections and databases, our results produced limited results,” which underscores the limited or non-existent presence of protein-related massive databases such as UniProt, InterPro, RCSB PDB, etc., in Russia. Through our literature survey, it became clear that protein research in Russia extends beyond the scope of this review, which is why we explicitly stated: “explaining that the full scope of protein research in Russia is beyond the limits of this manuscript.”

Bioinformatics education in Russia is gaining momentum and many universities and institutes have initiated bioinformatics programmes at different levels, as we discussed in our review article. To this regard APPENDIX 1 presented in our article presents “selected” Russian laboratories or institutes who are working in the field of bioinformatics or allied disciplines including number 37 in the APPENDIX 1 “Faculty of Bioengineering and Bioinformatics at Lomonosov Moscow State University” and not every single one. Bioinformatics education is clearly a topic that merits a dedicated article, which is currently being prepared.

The “mysterious person” is indeed Peter the Great, and the text (AН CCCP in Russian), as we indicated in our article, refers to the Academy of Sciences of the USSR. In its simplified form, even the website of the Presidential Library, The Administrative Directorate of the President of the Russian Federation mentions “The Academy was established by order of Peter I on January 13 (24), 1724” (https://www.prlib.ru/en/collections/1320214). However, for the understanding of the readers of the article, the historical name of the academy was the St. Petersburg Academy of Sciences (or the Imperial Academy of Sciences), which was founded by the decree of Peter the Great (1724) and officially opened in December 1725. Later, it was reorganized as the Academy of Sciences of the USSR (1925-1991), and restored to its present name (Russian Academy of Sciences) in 1991 (https://publications.hse.ru/pubs/share/folder/rkk64gho9q/69395061.pdf; https://www.prlib.ru/history/618924). For the typo, or any other instance, a corrigendum will be published. Regarding George Gamow’s work, the discussion is indeed broader, and there has been ongoing debate about his contribution to the genetic code. Since our article primarily focused on bioinformatics research, we did not delve into this topic in detail, but instead cited a relevant work that explores it further (see reference 33 in the published review article). The cited work states: ‘Firstly, he asserted that one could work out the code solely from a knowledge of the sequence of bases in a DNA molecule and the sequence of amino acids in the protein that is coded…’ and ‘Secondly, he simplified the problem even more by reducing it to a numerical exercise. There were four bases in DNA, he pointed out, and 20 amino acids in proteins (The figure of 20 was a bold guess that turned out to be correct, though the actual 20 are not the same as those that Gamow listed)’ [6]. Based on this context, we chose to describe his work as having ‘optimistically proposed a fairly precise’ model. Regarding the references, (references 1, 2, and 4 in our published article) are indeed relevant. These references establish the definition of bioinformatics at the beginning of our article, providing clarity and basis on the subject to the readers. Similarly, concerning the references related to large-scale plant genomics projects, the critique misconceived our statement. Here, we pointed out the scale and intensity of international genomics initiatives and noted that such projects have not yet been realized in Russia. How does this relate to Russian bioinformatics? Only when a large-scale genomics project is initiated could it provide researchers with the opportunities and data necessary for developing related tools and databases for handling, storing, and analyzing this data. This is precisely what we specified in the following sentence: “…have not yet been realized. Lack of such large-scale projects (and data) might be a factor related to the nonavailability and underdevelopment of plant omics analysis tools in Russia.”

Information Box 1 is indeed connected to bioinformatics, and through this example, we indicate how related industries contributed towards the field. Here, we would like to highlight our statement: “Therefore, through this example, it is understandable that the pharmaceutical and healthcare industries promoted research in the field of bioinformatics.” This perspective was also emphasized during the peer review process, where one anonymous reviewer suggested providing further context on the contributions of different research efforts in response to the pandemic. The reviewer wrote: “I would like to suggest providing more specific details regarding how researchers have tackled the SARS pandemic. This could include information about the research institutions or teams involved in these studies, the methods and techniques employed, as well as the achievements they have made. By including these additional details, readers will gain a better understanding of the contributions made by various researchers in response to the pandemic.” Nevertheless, given the current global context, COVID-19 serves as a recent example of how researchers across scientific fields—including bioinformatics—have played an essential role in supporting humanity’s efforts. It is important to note that Information Box 1 cannot be considered a full-length article, as the breadth of the topic, even within the Russian context, requires a dedicated review.

In conclusion, we greatly appreciate the feedback and recognize the areas for further refinement. However, we respectfully maintain that the central themes of our article, particularly on bioinformatics research in Russia, have been brought to attention. While there may be disagreements about the inclusion of specific studies or institutions, our focus was on providing an insightful overview of major research directions, rather than detailing individual contribution. Overall, we would like to emphasize that when presenting general definitions, stating global facts, or making comparative statements, citing relevant and authoritative literature is crucial. In this regard, articles from non-Russian affiliations are indeed pertinent to the topic at hand. As active editors and reviewers, the authors understand the peer-review process and its importance in maintaining scholarly rigor.

## Data Availability

There are no new data associated with this article.
